# Knowledge, Attitude and Practices Towards Treating Pregnant Patients Among Dental Professionals in Russia

**DOI:** 10.3390/dj13100457

**Published:** 2025-10-06

**Authors:** Ksenia Babina, Maria Polyakova, Irina Makeeva, Inna Sokhova, Anna Mikheikina, Alexandr Zaytsev, Nina Novozhilova

**Affiliations:** 1 Department of Therapeutic Dentistry, Federal State Autonomous Educational Institution of Higher Education I.M. Sechenov First Moscow State Medical University of the Ministry of Health of the Russian Federation (Sechenovskiy University), 119991 Moscow, Russia; polyakova_m_a_1@staff.sechenov.ru (M.P.); makeeva_i_m@staff.sechenov.ru (I.M.); sokhova_i_a@staff.sechenov.ru (I.S.); mikheykina_a_m@staff.sechenov.ru (A.M.); novozhilova_n_e@staff.sechenov.ru (N.N.); 2 Institute of Foreign Languages for Professional Purposes, Federal State Autonomous Educational Institution of Higher Education I.M. Sechenov First Moscow State Medical University of the Ministry of Health of the Russian Federation (Sechenovskiy University), 119991 Moscow, Russia; zaytsev_a_b@staff.sechenov.ru

**Keywords:** dental care, dentists, health knowledge, attitudes, practice, pregnant women

## Abstract

**Objectives**: The study assessed knowledge, attitude, and practices of dentists towards treating pregnant patients. **Methods**: It was based on a cross-sectional, electronically administered survey of a convenience sample of Russian dentists conducted between March and April 2025. Our questionnaire was developed based on international guidelines and previously published surveys and validated through expert review and pilot testing. It contains four sections: Demographics, Knowledge, Attitude, and Practice. The overall knowledge and attitude scores were calculated and compared across subgroups based on gender, specialty, and experience using the Kruskal–Wallis and Dunn’s tests. Spearman’s coefficient was calculated to reveal pair-wise correlations between knowledge, attitude, and age of the participants. **Results**: Overall, 403 dentists completed the questionnaire. The majority of the participants (53%) demonstrated fair knowledge about providing dental care throughout pregnancy. Female dentists had a significantly higher median knowledge score compared with that of males; orthodontists and prosthetic dentists showed lower knowledge scores than other specialists. All participants demonstrated a positive attitude toward treating pregnant patients; however, a considerable number of dentists agreed that they do not feel comfortable treating pregnant women and prefer not to do that. A large majority of dental practitioners (83%) indicated that they had treated pregnant women. Liability concerns (42%) and a lack of knowledge (24%) were reported as the main barriers for providing dental care to pregnant patients. **Conclusions**: It can be concluded that insufficient knowledge influenced by gender and specialty is one of the key barriers to the dental treatment of pregnant women, despite generally positive attitudes of the practitioners.

## 1. Introduction

Pregnancy is a delicate condition that involves multiple physiological (i.e., physical, behavioral, hormonal, and immunological) changes for the proper development of the fetus [1,2,3]. Such changes affect various organs and systems [4] including the oral cavity [5,6].

Oral diseases are among the most common health problems experienced by pregnant women [7]. The most prevalent oral diseases in pregnancy are periodontal disease (gingivitis, periodontitis, and pyogenic granuloma), caries, and enamel erosion [1,4,6,7,8,9,10,11,12,13,14,15].

Oral health throughout pregnancy is essential for women’s general health and quality of life [16], as well as for normal fetal development [6,10]. For example, growing evidence suggests that periodontal disease may be a potential risk factor for adverse pregnancy outcomes [4,6,10,17,18,19]; therefore, a delay in dental treatment might adversely affect both the maternal and fetal health [20]. It is important to acknowledge that pregnancy itself is not a reason to postpone dental treatment, but its management requires a profound understanding of the condition and deep knowledge of appropriate treatment approaches [4].

It is widely accepted that prenatal dental treatment can be safely performed at any stage of pregnancy [1,9,21,22]. This may imply local anesthesia, radiography, and administration of local and systemic medications [23]. The FDA classifies each medication into 1 of 5 pregnancy risk categories (A, B, C, D, or X) depending on the safety of its use in pregnant women [24]. There are very few drugs classified as category A; therefore, category B medicaments are commonly prescribed during pregnancy. These drugs have not demonstrated a fetal risk in animal studies; thus, medications in this class are generally considered safe [24].

In general, there is no evidence that exposure to dental care, particularly local anesthesia, during pregnancy (including the first trimester) may be associated with an increased risk for major anomalies of the fetus [25]. For example, the most commonly used anesthetic Lidocaine belongs to category B according to the FDA pregnancy risk classification [26]. If dental treatment requires antibiotic intake, there are some medications considered safe in pregnancy, including penicillins and cephalosporins (group B). Metronidazole is also classified in group B in second and third trimesters; however, its use is prohibited in the first trimester due to its teratogenic effects [26]. When it comes to NSAIDs, paracetamol or acetaminophen is categorized in group B according to the FDA classification and it is the most commonly used painkiller for pregnant women. Other common NSAIDs such as ibuprofen and ketoprofen also belong to category B in the first and second trimesters, but move to category D in the third trimester due to unfavorable effects on pregnancy course [26]. Apart from the use of different medications, many dental procedures require radiographic examination, which is considered safe in pregnant patients if proper shielding during exposure is provided [21,27]. However, pregnant women should only be exposed to X-rays if it is necessary to make a diagnosis, while routine X-rays should be postponed until after delivery [28].

Despite the fact that multiple international guidelines confirm the possibility of performing dental treatment in pregnant patients, there are common and persistent myths among dental professionals regarding dental treatment safety and possible adverse effects [6,29]. The most common unfavorable beliefs are that painkiller and antibiotic use, local anesthesia, radiographic examination, and endodontic and periodontal treatments are contraindicated in pregnancy [4,9,20,30,31]. These beliefs are mainly related to a lack of knowledge and poor understanding of indications, contraindications, and precautions of dental therapies in pregnancy [1,29]. As a result, dental practitioners are often hesitant to treat pregnant women and prefer to postpone dental treatments to the period after delivery [1,4,7,10,20]. Given the fact that a delay in dental treatment may adversely affect maternal and fetal health, it is crucial to increase awareness on the topic. Therefore, the aim of our study was to assess knowledge, attitude, and practices of dentists towards treating pregnant patients.

## 2. Materials and Methods

The ethical approval for the study was granted by the Ethics Committee of FSAEI HE I.M. Sechenov First MSMU of MOH of Russia (Sechenovskiy University), Moscow, Russia (Protocol No. 05-25, 12 March 2025). This study conducted between March and April 2025 was based on a cross-sectional, electronically administered survey of a convenience sample of dentists practicing in Russia. Participation was voluntary and anonymous, and the submission of a completed questionnaire implied informed consent for the participation in the study and publication of the results (in aggregate form). The researchers ensured the privacy and confidentiality of all the information collected.

Questionnaire development included review of the literature and international guidelines on providing dental treatments to pregnant women [1,21,24,26]. Most of the survey questions were adapted from previously published validated questionnaires on the topic [4,5,7,17,32,33,34,35] and translated into the Russian language by a professional linguist. The draft questionnaire was reviewed by 5 experts in the field to establish content validity and then piloted with 30 random dental professionals to check the clarity of the statements and to assess the time needed to complete the form. The survey for piloting was distributed via professional groups in social media platforms (Telegramm and VK). Respondents’ feedback was used to refine wording, content, and length of survey. The final survey was disseminated using Google forms via social media platforms for dentists. Sampling was continued until the end of the day, when predicted sample size was achieved.

The targeted samples included practitioners working in different fields of dentistry in Russia. The total number of the study population was estimated to be around 71,000 (a number of dental practitioners according to Federal State Statistics Service). A minimum sample size of 383 respondents was calculated assuming a 95% CI and a margin of error of 5% using the following formula:

$$Sample\;size=\;\frac{\frac{z^{2}\times p\left(1-p\right)}{e^{2}}}{1+\left(\frac{z^{2}\times p\left(1-p\right)}{e^{2}N}\right)}$$

where

*z* (z-score) = 1.96 (95% CI);

*p* (standard deviation) = 0.5;

*e* (margin of error) = 0.05;

*N* (population size) = 71,000 [36,37].

The 15-min 36-item questionnaire was divided into the following four sections: Demographics (5 questions), Knowledge (17 questions), Attitude (5 questions), and Practice (9 questions). One additional question asked about the willingness to receive additional information on dental care during pregnancy.

In the Demographics section, dental professionals were asked about their age, gender, work experience, and specialization.

The Knowledge section contained questions regarding the possibility of providing dental care in different trimesters of pregnancy and special considerations on treatment and diagnostic approaches. There were 10 “Yes/No/I don’t know” questions, 2 multiple choice questions, 4 single choice questions, and 1 matrix type question. Each correct answer scored one. For the question asking about NSAIDs prescription, score 1 was assigned if participants correctly answered about the use of at least two medicaments in different trimesters. The maximum total score for the Knowledge section was 20. The overall knowledge score was considered poor if it was lower than 50% (<10 points), fair if it was between 50% and 75% (≥10 and ≤15 points), and good if it was greater than 75% (>15 points).

The Attitude section contained questions asking about respondents’ personal opinion on the importance of dental care during pregnancy and willingness to treat pregnant women. The items were scored based on a five-point Likert scale (from ‘strongly agree’ to ‘strongly disagree’). The overall attitude score ranged from 5 (most negative attitudes) to 25 (most positive attitudes). The total attitude score was regarded as positive if it was higher than 50% (>12 points) or negative if it was lower than 50% (≤12 points).

The Practice section contained questions assessing dental treatment practices and barriers for treating pregnant patients. There were two open-ended questions, two “Yes/No” questions, and two single choice and three multiple choice questions.

Data manipulation was performed through MS Excel version 16.93.1 and R software (version 4.4.1; R Foundation for Statistical Computing, Vienna, Austria) with the RStudio environment (version 2024.09.0+375, Posit, PBC, Boston, MA, USA) using “dplyr” and “rstatix” packages. Descriptive statistics were presented as counts (*n*) and percentages (%) for categorical variables and medians with interquartile ranges (IQR), as well as means and standard deviations (sd) for continuous variables. Differences among groups were evaluated using the Kruskal–Wallis test followed by Dunn’s post hoc test. Spearman’s rank coefficient was calculated to reveal pair-wise correlations between knowledge, attitude, and age of the participants.

## 3. Results

Table 1 shows the demographic characteristics of the survey respondents, in aggregate and by gender, specialty, and clinical experience. Overall, 403 dentists completed the questionnaire. Most study participants were mid-career females and worked as conservative and general dentists. The mean age of the participating dental professionals was 35 ± 11 years.

Table 2 provides the breakdown of overall knowledge and attitude scores according to demographic characteristics. The median knowledge score was 11. Only a small minority of participants (17%) demonstrated a good level of knowledge about providing dental care throughout pregnancy, while a significant proportion (30%) showed poor knowledge on the topic. Female dentists had a significantly higher median knowledge score compared with that of males (*p* < 0.001).

Table 3 illustrates the proportion of answers to knowledge questions. Roughly three fifths of the participants believed that it is safe to provide dental prophylaxis (66%) and local anesthesia (69%) throughout all stages of pregnancy, while only 43% and 35% believed that dental radiographs and periodontal care are safe. Interestingly, Articaine (73%) and Mepivacaine (35%) were thought to be the safest anesthetics, while Lidocaine was chosen only by 9% of the respondents. Also, the majority of the dentists (74%) believed that it is unsafe to use vasoconstrictors in pregnancy. Regarding the positioning of pregnant women in the dental chair in the third trimester, only a quarter correctly chose lying to the left.

Thirty-six percent and 34% of dental professionals thought that NSAIDs and antibiotics are strongly contraindicated during pregnancy. Additional questions were available to those respondents who answered that the aforementioned medicaments are not contraindicated in pregnant patients (*n* = 200). Penicillins (92%) and macrolides (26%) were regarded as the safest antibiotics, and macrolides were chosen as the best alternative to penicillins in patients allergic to them (72%). The majority of the dentists believed that metronidazole is contraindicated during all stages of pregnancy (70%).

Regarding the appropriate timing of safe NSAIDs administration during pregnancy (Table 4), all drugs were predominantly identified as safe for use during the second trimester. Acetaminophen was correctly considered the safest drug that may be prescribed in all trimesters. Ibuprofen was thought to be safe during the second trimester (72%); however, fewer respondents considered it safe in the first trimester (38%), with the same proportion of dentists (38%) incorrectly considering it safe during the third trimester. Ketorolac and nimesulide were correctly identified as contraindicated by a large proportion of participants (40% and 44%, respectively). However, for these same drugs, many respondents incorrectly identified the second trimester as a safe period (40% and 56%, respectively).

Table 5 shows the distribution of responses to “attitude” questions among dental professionals.

All participants demonstrated a positive attitude toward treating pregnant patients (overall attitude score > 12). There were statistical differences in attitude scores noted by gender, specialty, and years in practice (Table 2). Substantial controversy was found in the attitudes towards limiting dental care for pregnant women to emergencies, with 45% of respondents disagreeing and 29% agreeing with this statement. A considerable number of dentists agreed that they do not feel comfortable treating pregnant women and prefer not to do that (38% and 26%, respectively).

However, a large majority of dental practitioners (83%) indicated that they had treated pregnant women (Table 6). Liability concerns (42%) and a lack of knowledge (24%) were reported as the main barriers for providing dental care to pregnant patients.

Pulpitis (49%), caries (44%), and gingivitis (43%) were mentioned as the most common reasons to visit a dental office. The anesthetics of choice were Articaine (84%) and Mepivacaine (13%). Only 17% of participating dentists prescribed antibiotics during pregnancy, mostly Penicillins (78.6%) and Macrolides (14.3%).

Table 7 shows the frequencies and timing of different dental treatments provided to pregnant women. Among the most common procedures were anesthesia (77%), routine prophylaxis (75%), and emergent restorative treatment (74%). All treatments were predominantly provided during the second trimester.

A vast majority of the participants (96%) were interested in receiving additional information on the treatment of pregnant patients. The respondents preferred to receive the information from printed materials (66%) and online courses (64%) (Table 6).

**Figure 1:**
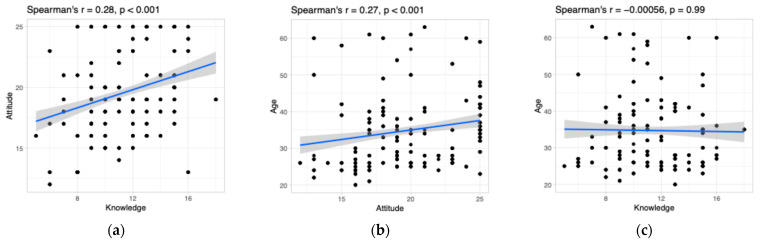
The correlation analysis: (**a**) the correlation between attitude and knowledge; (**b**) the correlation between attitude and age; (**c**) the correlation between knowledge and age. Each dot represents a pair of datapoints for two variables; the blue line represents a trend of correlation.

The results of the correlational analysis are shown in Figure 1. A weak yet significant positive correlation was found between attitudes and knowledge scores and between attitude and participants’ age. There were no statistical associations between knowledge and participants’ age.

## 4. Discussion

In this study, we assessed knowledge, attitude, and practices of dentists towards providing dental care in pregnancy. We found evidence of hesitancy in treating pregnant women in a quarter of surveyed dentists as well as inappropriate limitation of the procedures that can be safely performed throughout pregnancy. These findings may be mainly attributed to their insufficient knowledge on the topic.

Only 17% of the participants demonstrated good knowledge about providing dental care throughout pregnancy; the median knowledge score was 11 out of 20. These findings corroborate those reported in previous studies [7,10,17,35,38].

Regarding the ideal period for treating pregnant patients, a large majority of the respondents (89%) agreed that the second trimester is the safest. These findings are in agreement with those reported in the surveys by AlHalal et al. (70%) [4] and Pa Costa et al. (74%) [35]. However, a review by Pontes Vieira et al. revealed that dental professionals had doubts about the optimal gestational period for treatment [39].

According to Oral Health Care During Pregnancy: A National Consensus Statement (2012), emergency care should be provided at any time during the pregnancy [40]. In our study, 99% of respondents answered that emergency dental treatment can be performed, while dental prophylaxis and periodontal care were considered safe by 66% and 35% of the participants, respectively. In a study by Schramm et al., 86% of dental hygienists believed that dental hygiene services should not be limited to the second trimester. Moreover, the respondents indicated that women may receive prophylaxis (96%), emergency care (93%), periodontal treatment (76%), and restorative care (62%) throughout pregnancy [33]. According to Huang et al., 59.3% of dental faculty members agreed that pregnant women should receive not only emergency but also routine dental treatment [5]. In a study by Ibhawoh et al., a vast majority of the respondents (92%) considered endodontic treatment safe throughout pregnancy [32]. On the other hand, in a survey by Pa Costa et al., 66% of the participants responded that elective restorative treatment should be delayed until after pregnancy, and 10% believed that pregnant women should get dental treatment only in case of emergencies [35].

Three fourths of the dentists (74%) surveyed in our study knew that uncontrolled periodontitis can cause negative birth outcomes. In the study by AlHalal et al., 99% of dentists reported that periodontal disease can cause preterm birth, low birth weight, and gestational diabetes [4]. In contrast, George et al. reported that a relatively low proportion of dental specialists correctly identified the possible association between periodontal disease and various birth outcomes (18.9–65.9%) [7]. In the study by Pa Costa et al., near a quarter of the surveyed dentists were not sure about the correlation between periodontitis and elevated risk of low birth weight or preterm birth; however, 77% and 69%, respectively, believed that periodontal disease may increase the risk of the aforementioned outcomes [35].

In our study, 77% of the respondents believed that obstetricians should always be consulted prior to treating a pregnant woman. Similarly, in the study by AlHalal et al., 99% of the participants believed that it is necessary to consult with an obstetrician prior to treating pregnant women [4]. However, in the study be Huang et al., only about a third of respondents (30%) agreed that obstetricians should always be consulted before dental treatment [5]. George et al. found that dentists who believed that a consent from obstetricians is necessary tended to delay dental visits until after delivery [7]. It should be noted that a consultation with an obstetrician is not required for most of dental treatments in healthy pregnant women [39].

In our survey, we asked about an optimal position for treating pregnant women during the third trimester; only 25% of the respondents chose lying to the left. AlHalal et al. reported that near half of the participants (46%) correctly answered that the optimal position is lying to the left [4]. An incorrect position (lying to the right or supine) can increase the risk of vena cava syndrome or supine hypotensive syndrome development [41]. Vena cava runs along the right side of the vertebral column; in the supine or right-inclined positions, the uterus may compress the inferior vena cava leading to decreased venous return centrally and limited blood flow to the placenta. In severe cases, this may lead to unconsciousness and even morbidity and mortality for the mother and fetus [42,43,44].

One more issue associated with doubts and fears among dentists is the use of X-ray during pregnancy [39]. Prasad et al. and Razi et al. found a satisfactory level of knowledge regarding awareness on the topic [20,45]. In our study, 43% of dentists answered that, if required, X-ray may be performed in any of the trimesters. Previous surveys reported that 43% [5] and 75% [35] of the dental practitioners considered dental radiographs safe in pregnant women.

As regards attitude, almost all participating dentists agreed that maintaining oral health during pregnancy is important and that oral health checkups should be a routine component of pregnancy monitoring. Similarly, in a study by George et al., a vast majority of surveyed dentists considered maintaining oral health during pregnancy important (99.5%) and believed that pregnant women should visit a dentist early in pregnancy (98.9%) [7]. Similar data were reported in other studies [5,38].

A large majority of the surveyed dentists (83%) indicated that they had treated pregnant women. These results are similar to those reported by Pa Costa et al., who found that 87% of dental professionals provided dental treatment to pregnant patients, while 48% provided comprehensive treatment [35]. In the study by AlHalal et al., 61% had provided dental treatment to pregnant women [4].

At the same time, the respondents of our survey reported treating pregnant women infrequently: around a half of them reported seeing a pregnant woman once in 6–12 months. This rate is relatively lower than that found in previously published studies. For example, according to Lee et al., the surveyed dentists in aggregate had two to three visits of pregnant patients per week [17], while according to George et al., 87% of the dentists treated at least one pregnant woman per month [7]. At the same time, 52% of the respondents sometimes recommended pregnant patients to delay dental visits until after delivery [7].

We observed differences in the care dentists would offer to pregnant patients. In our study, more than half of the participants reported that they always advise pregnant women on oral health irrespective of the reason of visit. Previous studies have reported a greater proportion of dental professionals always counselling and providing health instructions prior to dental care in pregnancy [4,7]. The most common reasons to visit a dental office reported by our respondents included pulpitis (49%), caries (44%), and gingivitis (43%). According to Razban et al., pregnant women mentioned the following reasons to consult their dentist: routine check-ups (60%), “infection” (20%), and “gingival bleeding” (17%). We found that anesthesia (77%), routine prophylaxis (75%), and emergent restorative treatment (74%) were the most common procedures provided to pregnant women, while prosthetic and periodontal treatments were relatively uncommon. Pa Costa et al. reported that counseling and preventive services were the most common dental services provided to pregnant women [35]. Regarding the types of treatments performed in pregnancy, Lee et al. found that 56% of dentists relatively often performed tooth extraction, 29% performed composite resin restorations, and 41% provided non-surgical periodontal treatment. At the same time, only 27% of dentists indicated that they often or sometimes gave injectable local anesthesia before dental treatment [17]. According to Ibhawoh et al., a large majority (77.0%) of dental professionals would perform endodontic treatment in a pregnant woman [32].

The participants of our survey preferred to provide all treatments during the second trimester. Lee et al. found that the surveyed dentists would not perform composite restorations during the first (66%), second (25%), or third trimesters (43%). Similarly, in their study, the dentists replied that they would not perform nonsurgical periodontal treatment in any trimester (57%, 22%, and 46%) [17].

Many dental procedures may require radiographic examination. In our study, more than half of the dentists indicated that they do not use radiography in pregnant patients, while more than a third of the respondents used this diagnostic method in the second trimester. In the survey by George et al., 13% of dentists answered that they always undertake routine single periapical radiographs in pregnant women [7]. AlHalal et al. reported that radiovisiography was the most commonly used radiographic modality (41.5%), whereas cone-beam computed tomography (CBCT) was chosen by only one participant [4]. On the other hand, Patel et al. found that 35% of endodontists would prescribe a CBCT scan for a pregnant patient [46]. The FDA recommends to postpone routine dental X-ray examination until after delivery [28]. On the other hand, it should be emphasized that avoiding necessary radiographic imaging may result in unnecessary delays in diagnosis and treatment, which could lead to complications for the mother and fetus [47,48].

Regarding the pharmacological management of pregnant women, there is a controversy in dentists’ prescriptions [39]. In our study, of the respondents who reported treating pregnant women, 23% do not provide local anesthesia. Most participants indicated that they used local anesthesia in the second trimester; the anesthetics of choice were Articaine (84%) and Mepivacaine (13%). A relatively high frequency of Mepivacaine use may be explained by a belief that it is unsafe to use vasoconstrictors in pregnant patients (74%). It should be mentioned that dental procedures with local anesthesia are safe, including treatments during the first trimester [25]. Moreover, vasoconstrictors may increase the safety of anesthesia by delaying the entry of anesthetic into the bloodstream of the mother and fetus, thus leading to a gradual increase in its systemic concentration [49]. When properly administered, epinephrine is considered a safe and effective vasoconstrictor in pregnancy [50].

In the study by AlHalal, lidocaine hydrochloride with adrenaline was chosen by more than half of the dentists [4] A relatively low level of lidocaine use in our survey is consistent with Russian clinical guidelines, which recommend Articaine for pregnant patients. Another factor is that Lidocaine is not commercially available in pre-mixed dental cartridges with a vasoconstrictor. Therefore, there is a need for manual, chairside preparation that may be perceived as time-consuming and unsafe.

In our survey, few respondents reported prescribing antibiotics to pregnant women; the selected medications were limited to penicillins and macrolides, which is consistent with current clinical guidelines [1,26]. On the other hand, despite its classification as an FDA Category B drug for use in the second and third trimesters, metronidazole was incorrectly considered unsafe throughout all stages of pregnancy by 70% of dentists. Our findings corroborate those reported by Aragoneses et al., who found that the antibiotics of choice for pregnant women were amoxicillin and amoxicillin-clavulanate, while less frequently prescribed antibiotics were azithromycin, clindamycin, and metronidazole. The second trimester was considered the safest for antibiotic administration followed by the third trimester [16].

Regarding the appropriate timing of safe NSAIDs administration during pregnancy, a considerable uncertainty was revealed, with many respondents selecting “I don’t know” (12–20% for different medicaments). Acetaminophen was correctly considered the safest drug that may be prescribed in all trimesters. These results are in agreement with those reported by AlHalal, who found that more than half of the dentists chose acetaminophen (68%) as a pain-killer and considered it safe [4]. On the other hand, 38% of the respondents in our survey believed that ibuprofen can be prescribed during the third trimester, while the FDA recommends avoiding its use as well as the use of other NSAIDs at 20 weeks of pregnancy or later [51]. All in all, the results indicated serious knowledge gaps regarding NSAIDs use throughout pregnancy.

In our study, 26% of the respondents answered that they would prefer not to treat pregnant women, and 38% of the respondents answered that they did not feel comfortable treating pregnant patients. Similarly, 41% of the respondents in the survey by Huang’s et al. preferred not to treat women during pregnancy, and half of the respondents felt uncomfortable when treating pregnant women [5]. Lee at al. reported that more than half of the dentists indicated they were hesitant to perform routine dental care in pregnancy, and three quarters were hesitant to provide even emergency treatment [17]. In a study by Schramm et al., 90% of dental hygienists expressed willingness to provide care throughout pregnancy [33].

In our survey, the aforementioned attitudes of dentists towards treating pregnant women were reflected in their everyday clinical practice. Almost 17% of the respondents stated that they refrain from treating pregnant women; the most common reasons were liability concerns, personal discomfort, and lack of knowledge on the topic. Similarly, Pa Costa et al. stated that one in five dentists avoids providing dental care for pregnant women due to a lack of knowledge [35]. In the study by AlHalal et al., near a quarter of the participants believed that they did not have sufficient knowledge about dental treatment of pregnant women [4]. In our study, 96% of the dental practitioners stated that they would like to have more information about the principles of providing dental care in pregnancy, with online courses and printed materials chosen as the preferred sources of information. A number of previous surveys have concluded that dental professionals expressed the need for continuous education and clear guidelines [7,33,34]. Also, a lack of knowledge has been previously reported as one of the main barriers for providing dental care to pregnant patients [5,7,17,35].

Given that insufficient knowledge is one of the most important barriers to providing dental care for pregnant women, it is crucial to reveal the factors that influence dental practitioners’ knowledge and attitudes. We found that gender and specialty significantly impacted knowledge scores, while professional experience did not. Orthodontists and prosthetic dentists showed lower knowledge than other specialists. This may be due to the fact that different specialties are unequally involved in providing dental care to pregnant patients. We found higher knowledge scores among female dentists in our study. It can be hypothesized that this may stem from their personal experiences of pregnancy and, thus, higher empathy towards pregnant patients resulting in more accurate and thorough studying of clinical protocols and pharmacotherapeutic options.

The lack of association with experience suggests that knowledge is not passively accumulated over time, but rather dentists require continuing education to be aware of the current scientific developments. Similarly, we found no significant correlation between knowledge and participants’ age. These findings are in contrast to the study by Razi et al., who revealed an inverse correlation between dentists’ age and their level of awareness [20]. On the other hand, AlHalal et al. found that a significant increase in self-perceived knowledge with age did not result in different clinical behaviors, as no statistically significant differences were observed among the different age groups and their responses to the attitude and practice questions [4]. This finding is corroborated by Aragoneses et al., who also concluded that there were no statistically significant differences among the different age groups and their responses to the attitude and practice questions on antibiotic prescriptions to pregnant and breastfeeding women [16].

Overall, despite the existence of multiple international guidelines confirming the possibility of dental treatment in pregnant patients, Russian dentists were found to refrain from treating pregnant women, unnecessarily limiting the number of procedures provided. Further research could focus on localizing existing antenatal oral healthcare guidelines, recommendations, and consensus statements, including, but not limited by, the following list:

- FDA Medications Guides [26];
- ADA’s recommendations [21];
- ACOG’s Guidelines for Diagnostic Imaging During Pregnancy and Lactation [27];
- Oral Health Care During Pregnancy: A National Consensus Statement [40].

Also, efforts should be directed towards developing and providing adequate training on the medical and medicolegal aspects of dental care during pregnancy.

This study suffers from some limitations. First, convenience sampling covers only the individuals who use the internet and social platforms and excludes the offline population. Moreover, 90% of the respondents were females. Next, self-reported data of the survey may be prone to subjectivities and various response biases. Finally, biases may be related to the respondent’s access to the internet, particularly when answering knowledge questions.

## 5. Conclusions

Within the limitations of our study, it can be concluded that the overall knowledge on the subject was insufficient. Dental professionals in Russia tend to refrain from treating pregnant women, thus unnecessarily limiting the number of procedures provided (a lack of knowledge and liability concerns were mentioned by the participants as key barriers to the provision of treatment to pregnant patients). Further efforts should be focused on adapting existing guidelines to local contexts and developing and providing adequate training on the medical and medicolegal aspects of dental care during pregnancy.

## Figures and Tables

**Table 1:** Participants’ demographic characteristics.

Sociodemographic Data	Characteristics	Number (%)
Gender	Male	40 (10.0)
	Female	363 (90.0)
Age	Mean (SD)	34.7 (10.7)
	Median (IQR)	33 (26; 40)
Specialty	Conservative dentist	173 (42.9)
	Dental surgeon	43 (10.7)
	Orthodontist	72 (17.9)
	Prosthetic dentist	14 (3.5)
	General dentist	80 (19.9)
	Maxillo-facial surgeon	4 (1.0)
	Other	17 (4.2)
Years of clinical experience	<2	80 (19.9)
	2–5	75 (18.6)
	6–10	72 (17.9)
	11–20	108 (26.8)
	21–30	40 (9.9)
	>30	28 (6.9)
	<2	80 (19.9)

IQR—interquartile range; SD—standard deviation.

**Table 2:** Overall knowledge and attitude scores of the participants.

Sociodemographic Data	Knowledge (Maximum 20)		Attitude (Maximum 25)	
	Mean (SD)	Median (IQR)	Mean (SD)	Median (IQR)
Total	11.2 (2.7)	11 (9; 13)	19.5 (3.6)	19 (17; 23)
Male	9.3 (1.8)	9 (9; 11)	16.5 (2.5)	16.5 (16; 17)
Female	11.4 (2.8)	11 (9; 13)	19.8 (3.5)	19 (17; 23)
	Kruskal–Wallis chi-squared = 70.399, df = 6, *p* < 0.001 *		Kruskal–Wallis chi-squared = 34.359, df = 1, *p* < 0.001 *	
Conservative dentist	12.0 (2.6)	12 (10; 14) ^a^	20.3 (3.7)	20 (17; 24) ^a^
Dental surgeon	12.3 (2.3)	12 (11; 14) ^a^	18.5 (2.5)	19 (16; 19) ^ab^
Orthodontist	9.3 (2.0)	9.5 (8; 10) ^b^	18.5 (3.2)	18.5 (17; 21) ^b^
Prosthetic dentist	9.0 (2.1)	8 (7.25; 11.25) ^c^	17.3 (3.4)	16 (15.25; 17) ^b^
General dentist	11.0 (2.8)	11 (9; 12) ^bc^	19.6 (3.7)	20 (17; 23) ^ab^
Maxillo-facial surgeon	11 (0.0)	11 (11; 11) ^abc^	19 (0.2)	19 (18; 20) ^ab^
Other	10.9 (3.7)	10 (9; 15) ^abc^	20.2 (4.6)	20 (16; 25) ^ab^
	Kruskal–Wallis chi-squared = 22.139, df = 1, *p* < 0.001 *		Kruskal–Wallis chi-squared = 22.639, df = 6, *p* < 0.001 *	
Years of clinical experience				
<2	11.5 (2.9)	12 (9; 14)	17.3 (3.0)	17 (16; 19.25) ^a^
2–5	10.8 (2.9)	10 (9; 13)	18.9 (3.3)	19 (16.5; 21) ^b^
6–10	11.2 (3.1)	11 (9; 13)	20.9 (3.0)	20 (19; 23) ^c^
11–20	11.4 (2.3)	12 (9; 13)	20.7 (3.6)	20 (18; 25) ^c^
21–30	11.3 (2.5)	11 (10; 12)	19.2 (3.7)	18.5 (17; 23) ^abc^
>30	10.7 (3.1)	10 (8; 14)	19.7 (3.9)	20 (17; 24) ^bc^
	Kruskal–Wallis chi-squared = 4.6664, df = 5, *p* = 0.4579		Kruskal–Wallis chi-squared = 54.418, df = 5, *p* < 0.001 *	

* Differences are statistically significant; IQR—interquartile range; SD—standard deviation; df—degree of freedom, ^abc^ superscript letters indicate differences among the study groups.

**Table 3:** Distribution of participants’ responses to knowledge questions about dental care throughout pregnancy, *n* (%).

Item	Variants	Answers, *n* (%)
Emergency care can be provided to pregnant women throughout all stages of pregnancy.	Yes	399 (99.0)
	No	4 (1.0)
	I don’t know	0 (0)
The optimal trimester to provide routine dental care for a pregnant patient is…	First	12 (3.0)
	Second	359 (89.1)
	Third	28 (6.9)
	I don’t know	4 (1.0)
Obstetricians should always be consulted prior to treating a pregnant woman.	Yes	311 (77.2)
	No	84 (20.8)
	I don’t know	8 (2.0)
Pregnant women can safely receive routine preventive care throughout pregnancy.	Yes	264 (65.5)
	No	123 (30.5)
	I don’t know	16 (4.0)
Pregnant women can safely receive periodontal care (scaling/root planning) throughout all stages of pregnancy.	Yes	140 (34.7)
	No	207 (51.3)
	I don’t know	56 (14.0)
Active periodontitis may increase the risk of adverse pregnancy outcomes.	Yes	300 (74.4)
	No	47 (11.6)
	I don’t know	56 (14.0)
Pregnant women can receive local anesthesia throughout all stages of pregnancy.	Yes	276 (68.5)
	No	107 (26.5)
	I don’t know	20 (5.00)
What is the safest local anesthetic during pregnancy?	Mepivacaine	139 (34.5)
	Bupivacaine	32 (7.9)
	Lidocaine	36 (8.9)
	Articaine	296 (73.4)
It is unsafe to use a local anesthetic with a vasoconstrictor in pregnant patients.	Yes	299 (74.2)
	No	80 (19.9)
	I don’t know	24 (5.9)
Pregnant women can safely receive dental radiographs throughout all stages of pregnancy.	Yes	172 (42.7)
	No	203 (50.4)
	I don’t know	28 (6.9)
Administration of non-steroid anti-inflammatory drugs is contraindicated during pregnancy.	Yes	143 (35.5)
	No	200 (49.6)
	I don’t know	60 (14.9)
Administration of antibiotics is contraindicated during pregnancy.	Yes	135 (33.5)
	No	200 (49.6)
	I don’t know	68 (16.9)
What are the safest antibiotics during pregnancy? *n* = 200 *	Penicillins	184 (92.0)
	Macrolides	52 (26.0)
	Tetracyclines	0 (0)
	Flouroquinolones	16 (8.0)
	Lincosamides	16 (8.0)
What are the alternative antibiotics for pregnant women allergic to penicillin? *n* = 200 *	Amoxicillin clavulanates	64 (32.0)
	Macrolides	144 (72.0)
	Tetracyclines	0 (0)
	Flouroquinolones	32 (16.0)
	Lincosamides	28 (14.0)
Is metronidazole contraindicated during pregnancy? *n* = 200 *	Contraindicated during all stages of pregnancy	140 (70.0)
	Contraindicated in the first trimester	80 (40.0)
	Contraindicated in the second trimester	4 (2.0)
	Contraindicated in the third trimester	4 (2.0)
	Metronidazole is safe during all stages of pregnancy	40 (20.0)
What is the right position in the dental unit in the third trimester of pregnancy?	Inclined to the left	100 (24.8)
	Inclined to the right	72 (17.9)
	Supine position with legs slightly elevated	40 (9.9)
	Regular position	120 (29.8)
	No treatment should be provided during the third trimester of pregnancy	71 (17.6)

* Questions were available to the respondents depending on their previous answers, *n* indicates the number of participants who had the opportunity to answer this particular question.

**Table 4:** Distribution of the participants’ responses to the question regarding the use of NSAIDs during pregnancy.

Item	Timing of Safe NSAID Administration During Pregnancy *n* (%), *n* = 200 *				
	1st Trimester	2nd Trimester	3rd Trimester	Contraindicated	I Don’t Know
Metamizole	12 (6.0)	44 (22.0)	28 (14.0)	108 (54.0)	28 (14.0)
Aspirin (acetylsalicylic acid)	8 (4.0)	52 (26.0)	40 (20.0)	104 (52.0)	24 (12.0)
Ketoprofen	12 (6.0)	52 (26.0)	20 (10.0)	92 (46.0)	40 (20.0)
Ketorolac	12 (6.0)	40 (20.0)	20 (10.0)	100 (50.0)	40 (20.0)
Nimesulide	8 (4.0)	56 (28.0)	28 (14.0)	88 (44.0)	44 (22.0)
Ibuprofen	76 (38.0)	144 (72.0)	76 (38.0)	20 (10.0)	24 (12.0)
Acetaminophen	88 (44.0)	160 (80.0)	120 (60.0)	12 (16.0)	16 (18.0)

* These questions were available to 200 respondents who answered that the NSAIDs are not contraindicated in pregnant patients, NSAIDs—nonsteroidal anti-inflammatory drugs.

**Table 5:** Distribution of the participants’ responses to “attitude” questions among dental professionals, *n* (%).

Item	Respondents’ Answers *n* (%)				
	SA	A	N	D	SD
Maintaining oral health during pregnancy is important.	360 (89.3)	27 (6.7)	8 (2.0)	4 (1.0)	4 (1.0)
Oral health checkup should be a routine component of monitoring pregnant patients.	363 (90.0)	16 (4.0)	16 (4.0)	8 (2.0)	0 (0)
Pregnant women should receive only emergency dental care.	64 (15.9)	51 (12.7)	108 (26.8)	48 (11.9)	132 (32.7)
I do not feel comfortable treating pregnant women.	76 (18.85)	76 (18.85)	95 (23.6)	44 (10.9)	112 (27.8)
I prefer not to treat pregnant women.	51 (12.7)	52 (12.9)	120 (29.8)	32 (7.9)	148 (36.7)

SA—strongly agree; A—agree; N—neutral; D—disagree; SD—strongly disagree.

**Table 6:** Distribution of the participants’ responses to “practice” questions among dental professionals, *n* (%).

Item	Variants	Answers, *n* (%)
Do you treat pregnant patients?	Yes	336 (83.4)
	No	67 (16.6)
Why do you not provide dental care to pregnant women? *n* = 67 *	Do not feel I have enough knowledge	16 (23.8)
	I have no pregnant patients	35 (52.2)
	Do not feel comfortable treating pregnant patients	12 (17.9)
	I have liability concerns treating pregnant patients	28 (41.8)
How often do you provide dental treatments to pregnant patients? *n* = 336 *	At least once a month	44 (13.2)
	Once every 2 months	72 (21.4)
	Once every 6 months	120 (35.7)
	Once every 12 months	24 (7.1)
	Rarer than once every 12 months	76 (22.6)
Do you always advise pregnant women on oral health irrespective of the reason of visit? *n* = 336 *	Yes	263 (78.3)
	In high risk patients	32 (9.5)
	No	41 (12.2)
Based on your practice, what are the most common reasons for visiting dentists during pregnancy? *n* = 336 *	Enamel erosion	4 (1.2)
	Dentin hypersensitivity	62 (16.0)
	Oral mucosa diseases	64 (19.0)
	Gingivitis	144 (42.9)
	Caries	148 (44.0)
	Apical periodontitis	48 (13.1)
	Pulpitis	164 (48.8)
	Other	92 (27.4)
Which anesthetics do you use in pregnant women? *n* = 336 *	Articaine	281 (83.6)
	Mepivacaine	44 (13.1)
	Lidocaine	18 (5.4)
	Do not use anesthesia	9 (2.7)
Do you prescribe antibiotics to pregnant women? *n* = 336 *	Yes	56 (16.7)
	No	280 (83.3)
Which antibiotics do you prescribe to pregnant women? *n* = 336 *	Penicillin + beta-lactamase inhibitors	32 (57.2)
	Penicillins	12 (21.4)
	Macrolides	8 (14.3)
	Other	4 (7.1)
Are you interested in receiving additional information to enhance your knowledge on treating pregnant patients?	Yes, I would like to attend an offline workshop or seminar	12 (3.0)
	Yes, I would like to attend an online course (webinar)	259 (64.3)
	I would like to receive information from articles/education brochures/books	264 (65.5)
	No, I think I have enough knowledge on treating pregnant patients	16 (4.0)

* Questions were available to the respondents depending on their previous answers, *n* indicates the number of participants who had the opportunity to answer this particular question.

**Table 7:** The frequencies of different dental treatments provided to pregnant women, *n* (%).

Item	Dental Care Procedures Provided by the Dentists During Pregnancy and Their Timing (*n* = 336), *n* (%)			
	1st Trimester	2nd Trimester	3rd Trimester	Did Not Provide This Particular Procedure
Anesthesia	108 (32.1)	232 (69.0)	128 (38.1)	76 (22.6)
Radiography	32 (9.5)	120 (35.7)	52 (15.5)	196 (58.3)
Routine prophylaxis	128 (38.1)	208 (61.9)	120 (35.7)	84 (25.0)
Periodontal care (scaling and root planning)	16 (4.76)	52 (15.5)	36 (10.7)	272 (80.1)
Teeth extractions	40 (11.9)	76 (22.6)	56 (16.7)	236 (70.2)
Root canal treatment	72 (21.4)	172 (51.2)	100 (29.8)	128 (38.1)
Emergency surgery	44 (13.1)	76 (22.6)	48 (14.3)	240 (71.4)
Routine surgery	4 (1.2)	48 (14.3)	20 (6.0)	272 (80.1)
Prosthetic treatment (removable dentures)	0 (0)	20 (6.0)	12 (3.6)	304 (90.5)
Prosthetic treatment (fixed dentures)	0 (0)	16 (4.8)	12 (3.6)	308 (91.7)
Orthodontic treatment (initiation)	12 (3.6)	28 (8.3)	12 (3.6)	292 (86.9)
Orthodontic treatment (in progress)	48 (14.3)	48 (14.3)	40 (11.9)	256 (76.2)
Restorative treatment (emergency)	136 (40.5)	212 (63.1)	120 (35.7)	88 (26.2)
Esthetic teeth restoration	76 (22.6)	152 (45.2)	76 (22.6)	156 (46.4)
Teeth bleaching	0 (0)	12 (3.6)	16 (4.8)	300 (89.3)

## Data Availability

The datasets used and/or analyzed during the current study are available from the corresponding author upon reasonable request.

## Abbreviations

The following abbreviations are used in this manuscript:

FDA	Food and Drug Administration
NSAIDs	Nonsteroidal anti-inflammatory drugs
IQR	Interquartile range
SD	Standard deviation
df	Degree of freedom

## References

[1] Hyder T., Khan S., Moosa Z.H. (2023). Dental Care Of The Pregnant Patient: An Update Of Guidelines And Recommendations. J. Pak. Med. Assoc..

[2] Mockridge A., Maclennan K. (2019). Physiology of Pregnancy. Anaesth. Intensive Care Med..

[3] Lewis E. (2014). Exercise in Pregnancy. Aust. Fam. Physician.

[4] AlHalal H., Albayyat R.M., Alfhaed N.K., Fatani O., Fatani B. (2023). Knowledge, Attitude, and Practice Regarding Periodontal and Dental Diseases During Pregnancy Among Obstetricians and Dentists in King Saud University Medical City. Cureus.

[5] Huang S.S., Yang C., Cohen V., Russell S.L. (2022). What Factors Influence Dental Faculty’s Willingness to Treat Pregnant Women?. JDR Clin. Trans. Res..

[6] Rocha J.S., Arima L.Y., Werneck R.I., Moysés S.J., Baldani M.H. (2018). Determinants of Dental Care Attendance during Pregnancy: A Systematic Review. Caries Res..

[7] George A., Ajwani S., Bhole S., Dahlen H., Reath J., Korda A., Ng Chok H., Miranda C., Villarosa A., Johnson M. (2017). Knowledge, Attitude and Practises of Dentists towards Oral Health Care during Pregnancy: A Cross Sectional Survey in New South Wales, Australia. Aust. Dent. J..

[8] Zhou X., Zhong Y., Pan Z., Zhang J., Pan J. (2023). Physiology of Pregnancy and Oral Local Anesthesia Considerations. PeerJ.

[9] Favero V., Bacci C., Volpato A., Bandiera M., Favero L., Zanette G. (2021). Pregnancy and Dentistry: A Literature Review on Risk Management during Dental Surgical Procedures. Dent. J..

[10] Flores-Montalvo E., Córdova-Limaylla N., Ladera-Castañeda M., López-Gurreonero C., Echavarría-Gálvez A., Cornejo-Pinto A., Cervantes-Ganoza L., Cayo-Rojas C. (2023). Factors Associated with Knowledge about Pharmacological Management of Pregnant Women in Peruvian Dental Students: A Logistic Regression Analysis. BMC Med. Educ..

[11] Boyapati R., Cherukuri S.A., Bodduru R., Kiranmaye A. (2022). Influence of Female Sex Hormones in Different Stages of Women on Periodontium. J. Midlife Health.

[12] Sathish A.K., Varghese J., Fernandes A.J. (2022). The Impact of Sex Hormones on the Periodontium During a Woman’s Lifetime: A Concise-Review Update. Curr. Oral Health Rep..

[13] Cho G.J., Kim S.Y., Lee H.C., Kim H.Y., Lee K.M., Han S.W., Oh M.J. (2020). Association between Dental Caries and Adverse Pregnancy Outcomes. Sci. Rep..

[14] Velosa-Porras J., Rodríguez Malagón N. (2023). Prevalence of Dental Caries in Pregnant Colombian Women and Its Associated Factors. BMC Oral Health.

[15] Pecci-Lloret M.P., Linares-Pérez C., Pecci-Lloret M.R., Rodríguez-Lozano F.J., Oñate-Sánchez R.E. (2024). Oral Manifestations in Pregnant Women: A Systematic Review. J. Clin. Med..

[16] Aragoneses J., Suárez A., Rodríguez C., Algar J., Aragoneses J.M. (2021). Knowledge, Attitudes, and Practices among Dental Practitioners Regarding Antibiotic Prescriptions for Pregnant and Breastfeeding Women in the Dominican Republic. Antibiotics.

[17] Lee R.S.-Y., Milgrom P., Huebner C.E., Conrad D.A. (2010). Dentists’ Perceptions of Barriers to Providing Dental Care to Pregnant Women. Women’s Health Issues.

[18] Bobetsis Y.A., Ide M., Gürsoy M., Madianos P.N. (2023). Periodontal Diseases and Adverse Pregnancy Outcomes. Present and Future. Periodontol 2000.

[19] Wilson A., Hoang H., Bridgman H., Crocombe L., Bettiol S. (2022). Clinical Practice Guidelines and Consensus Statements for Antenatal Oral Healthcare: An Assessment of Their Methodological Quality and Content of Recommendations. PLoS ONE.

[20] Razi T., Bazvand L., Ghojazadeh M. (2011). Diagnostic Dental Radiation Risk during Pregnancy: Awareness among General Dentists in Tabriz. J. Dent. Res. Dent. Clin. Dent. Prospect..

[21] Pregnancy|American Dental Association. https://www.ada.org/resources/ada-library/oral-health-topics/pregnancy.

[22] Bao J., Huang X., Wang L., He Y., Rasubala L., Ren Y.F. (2022). Clinical Practice Guidelines for Oral Health Care during Pregnancy: A Systematic Evaluation and Summary Recommendations for General Dental Practitioners. Quintessence Int..

[23] Aliabadi T., Saberi E.A., Tabatabaie A.M., Tahmasebi E. (2022). Antibiotic Use in Endodontic Treatment during Pregnancy: A Narrative Review. Eur. J. Transl. Myol..

[24] Law R., Bozzo P., Koren G., Einarson A. (2010). FDA Pregnancy Risk Categories and the CPS: Do They Help or Are They a Hindrance?. Can. Fam. Physician.

[25] Hagai A., Diav-Citrin O., Shechtman S., Ornoy A. (2015). Pregnancy Outcome after in Utero Exposure to Local Anesthetics as Part of Dental Treatment: A Prospective Comparative Cohort Study. J. Am. Dent. Assoc..

[26] Medication Guides. https://dps.fda.gov/medguide.

[27] Guidelines for Diagnostic Imaging During Pregnancy and Lactation|ACOG. https://www.acog.org/clinical/clinical-guidance/committee-opinion/articles/2017/10/guidelines-for-diagnostic-imaging-during-pregnancy-and-lactation.

[28] The Selection of Patients for Dental Radiographic Examinations|FDA. https://www.fda.gov/radiation-emitting-products/medical-x-ray-imaging/selection-patients-dental-radiographic-examinations#patient_selection_criteria.

[29] Kamalabadi Y.M., Campbell M.K., Zitoun N.M., Jessani A. (2023). Unfavourable Beliefs about Oral Health and Safety of Dental Care during Pregnancy: A Systematic Review. BMC Oral Health.

[30] Wazir S.S., Ghosh S., Mahanta S., Shah R., Das A., Patil S. (2019). Knowledge, Attitude and Perception toward Radiation Hazards and Protection among Dental Undergraduates, Interns and Dental Surgeons—A Questionnaire-Based Cross-Sectional Study. J. Med. Radiol. Pathol. Surg..

[31] Alzamzami Z.T., Abulhamael A.M., Talim D.J., Khawaji H., Barzanji S., Roges R.A. (2019). Cone-Beam Computed Tomographic Usage: Survey of American Endodontists. J. Contemp. Dent. Pract..

[32] Ibhawoh L., Enabulele J. (2015). Endodontic Treatment of the Pregnant Patient: Knowledge, Attitude and Practices of Dental Residents. Niger. Med. J..

[33] Schramm S.A., Jacks M.E., Prihoda T.J., McComas M.J., Hernandez E.E. (2016). Oral Care for Pregnant Patients: A Survey of Dental Hygienists’ Knowledge, Attitudes and Practice. J. Dent. Hyg..

[34] Razban M., Giannopoulou C. (2020). Knowledge and Practices of Oral Health Care During Pregnancy: A Survey Among Swiss Dentists. Oral Health Prev. Dent..

[35] Pa Costa E.P., Lee J.Y., Rozler R.G., Zeldin L. (2010). Dental Care for Pregnant Women: An Assessment of North Carolina General Dentists. J. Am. Dent. Assoc..

[36] Babina K., Salikhova D., Polyakova M., Zaytsev A., Egiazaryan A., Novozhilova N. (2023). Knowledge and Attitude towards Probiotics among Dental Students and Teachers: A Cross-Sectional Survey. Dent. J..

[37] Mikheikina A., Novozhilova N., Polyakova M., Sokhova I., Mun A., Zaytsev A., Babina K., Makeeva I. (2023). Knowledge, Attitude, and Practice towards Chelating Agents in Endodontic Treatment among Dental Practitioners. Dent. J..

[38] Kloetzel M.K., Huebner C.E., Milgrom P., Littell C.T., Eggertsson H. (2012). Oral Health in Pregnancy: Educational Needs of Dental Professionals and Office Staff. J. Public Health Dent..

[39] Pontes Vieira D.R., Figueiredo de Oliveira A.E., Ferreira Lopes F., de Figueiredo Lopes e Maia M. (2015). Dentists’ Knowledge of Oral Health during Pregnancy: A Review of the Last 10 Years’ Publications. Community Dent. Health.

[40] National Maternal and Child Oral Health Resource Center (2012). Oral Health Care During Pregnancy: A National Consensus Statement.

[41] Naseem M., Khurshid Z., Khan H.A., Niazi F., Zohaib S., Zafar M.S. (2016). Oral Health Challenges in Pregnant Women: Recommendations for Dental Care Professionals. Saudi J. Dent. Res..

[42] Krywko D.M., King K.C. (2023). Aortocaval Compression Syndrome.

[43] Kim D.R., Wang E. (2014). Prevention of Supine Hypotensive Syndrome in Pregnant Women Treated with Transcranial Magnetic Stimulation. Psychiatry Res..

[44] Hemalatha V.T., Manigandan T., Sarumathi T., Aarthi Nisha V., Amudhan A. (2013). Dental Considerations in Pregnancy—A Critical Review on the Oral Care. J. Clin. Diagn. Res..

[45] Prasad M., Gupta R., Patthi B., Singla A., Pandita V., Kumar J., Malhi R., Vashishtha V. (2016). Imaging More Imagining Less: An Insight into Knowledge, Attitude and Practice Regarding Radiation Risk on Pregnant Women among Dentists of Ghaziabad—A Cross Sectional Study. J. Clin. Diagn. Res..

[46] Patel S., Brown J., Foschi F., Al-Nuaimi N., Fitton J. (2025). A Survey of Cone Beam Computed Tomography Use amongst Endodontic Specialists in the United Kingdom. Int. Endod. J..

[47] Toppenberg K.S., Hill D.A., Miller D.P. (1999). Safety of Radiographic Imaging during Pregnancy. Am. Fam. Physician.

[48] Yoon I., Slesinger T.L. (2023). Radiation Exposure in Pregnancy. StatPearls.

[49] Lee J.M., Shin T.J. (2017). Use of Local Anesthetics for Dental Treatment during Pregnancy; Safety for Parturient. J. Dent. Anesth. Pain Med..

[50] Urîtu A., Buciu V.B., Roi C., Chioran D., Serban D.M., Nicoleta N., Rusu E.L., Ionac M., Riviș M., Ciurescu S. (2025). Review of the Safety and Clinical Considerations of Vasoconstrictor Agents in Dental Anesthesia During Pregnancy. J. Clin. Med..

[51] Nonsteroidal Anti-Inflammatory Drugs (NSAIDs): Drug Safety Communication—Avoid Use of NSAIDs in Pregnancy at 20 Weeks or Later|FDA. https://www.fda.gov/safety/medical-product-safety-information/nonsteroidal-anti-inflammatory-drugs-nsaids-drug-safety-communication-avoid-use-nsaids-pregnancy-20.

